# Supplementary material for: Higher-order epistasis shapes natural variation in germ stem cell niche activity
Source: Nat Commun. 2023 May 17;14:2824. doi: 10.1038/s41467-023-38527-0 (PMC10192456; doi:10.1038/s41467-023-38527-0)
Supplement: Supplementary file 1 — Supplementary Information [file 41467_2023_38527_MOESM1_ESM.pdf]

Supplementary Information

Higher-order epistasis shapes natural variation in germ stem cell niche activity

Sarah R Fausett[1,2], Asma Sandjak[1], Bénédicte Billard[1], & Christian Braendle[1]

[1]Université Côte d'Azur, CNRS, Inserm, IBV, Nice, France
[2]Department of Biology and Marine Biology, University of North Carolina Wilmington, Wilmington, NC, USA

This PDF file includes:
Supplementary Figures 1 to 4
Supplementary Notes 1 to 12

Supplementary Figures

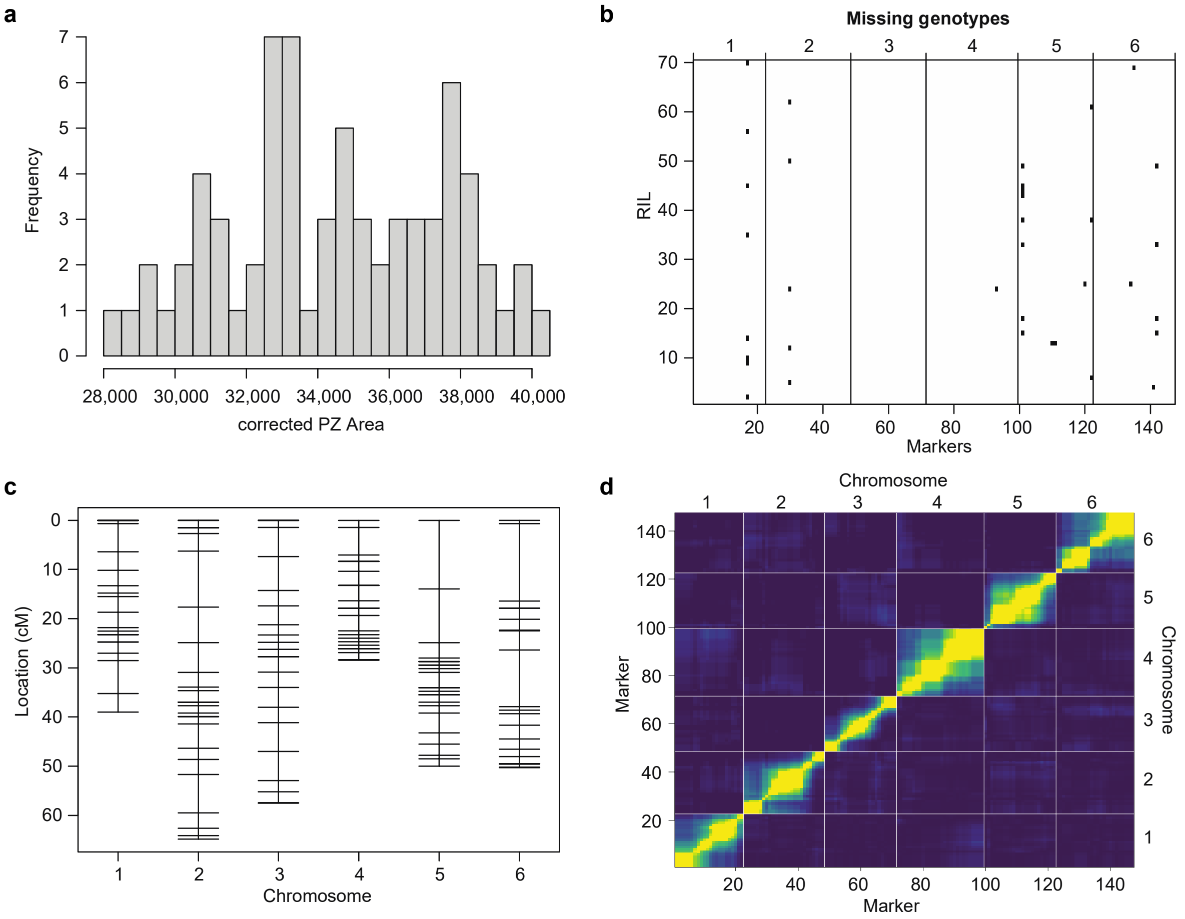

Supplementary Figure 1. Additional data related to RIL phenotyping and mapping.
a Phenotype distribution (PZ size) for all RILs. Corrected means for each RIL were calculated using correction factors derived from the least square means of a data set containing only the parental lines from each block (S3-S8). See Experimental Procedures and Supplementary Note 5 for more detail. **b** Missing genotypes for each RIL across all markers. **c** Genetic markers cover the entire genome. **d** Heat map of the recombination fractions (top) and LOD scores (bottom) for the genetic map, yellow corresponds to less recombination between markers while purple corresponds to more recombination between markers. LOD scores are derived by testing whether the recombination fraction equals 0.5, as per the est.rf() function in the R/qtl package. Data are provided as a Source Data file.

JU751cV del3.2 fwd1 / JU751cV del3.2 fwd2

TCCGTTATGACTCTATTGCTCCTAATCCCCCTTTTGTTTCCGGTTCGGCCTGAAACAAGAATAACAACAGGTGACTCGATA

ACTGTTAGGAAAATAAATTTTTTGGTCCCAATATTTCGGTATCCATTTCAAAACATCTTTTAAAATTGTCAAGACAGACAATT

JU751cV del3.2 F3

ACTTCTGCTTGAAATGAAGGACACATGTCCCCTCAATTCCTCATAAAACAAATAGAGGGGTGTCGAAATGATAAATGTTGC

G->A in repair

AAGTGAGGTGCAGATGGCAATGAAATTTATATATGTTT**CTTTCATTCCATTCTTGTGGGGCAATTTCAAACGTTTGATAAC**

HLH-2 site / JU751cV del3-2 F5/R5

TCACAGTGGTCTAGATTTGCAGAACATCTGTGTTAGGTCAAGCAGAGCGAGAATTTGAGCATTTGAACTTTAGGGTCC

G->A in repair / G->T in repair

TGGGGGGGGGAGGGGGGGGGGGGGGGGGGGGTGTCTAGACAGTCAGCGGCCCATAAGGGTTCATATGGTTTGACGG

TGCATTGAACTATTTTTTAAGGAAAAAATTGAGATAAAGGCCATTTTTCCAAGAAAGCATCTAGCTTTTTCAACTGACACTA

JU751cV del3-2 R4

CAAATTTCCCTATAACTTTCTGAAATTATCAGTGAAAAGTTGCCCAAATTTGAGACTTTAGCTAAAAATAGACAATTTTTTC

SNP C->A

CAAAACTTTGAGCGGCCATAACTTTTTTTTGAAAAACTTTCGGAACGTCTCATTACAAAATTTGGTTGCTTTGAGCCATTTT

GAATCCGAAAAAGTCTCAAAAAGCAAAGTCTTAAATTTCGGTACTCCACCTTTAAAAAAAATAGGGTTTCCGCCGAAATTT

CTGGGTTCCAATAATATCTGTTACCATTTCCTTGCTCTGCGACGAGCAGCCACCAGACAAAAATCTCCTAATACCCCTCTT

JU751cV del3.2 rev1

CATGCCTTTGTTCTTCCGGTTTGGCGGTCTGAAAAAGAGCACCCCTCCCGGCGGGGGGCTCTCGGTATCTGTTCCGCAA

JU751cV del3.2 rev2

AAACCGTCATATGGAGGGCTATCGGGAGACGGGAAGAGTTGGAAATACGGGAAATGGTGATG

Supplementary Figure 2. Portion of *lag-2* promoter sequence in JU1200 containing the 148 bp deleted in JU751. The deletion is bolded and underlined. The HLH-2 binding site[73], genotyping primers, CRISPR induced mutations, and SNPs are annotated[70,115]. See Supplementary Data 1 for primer sequences.

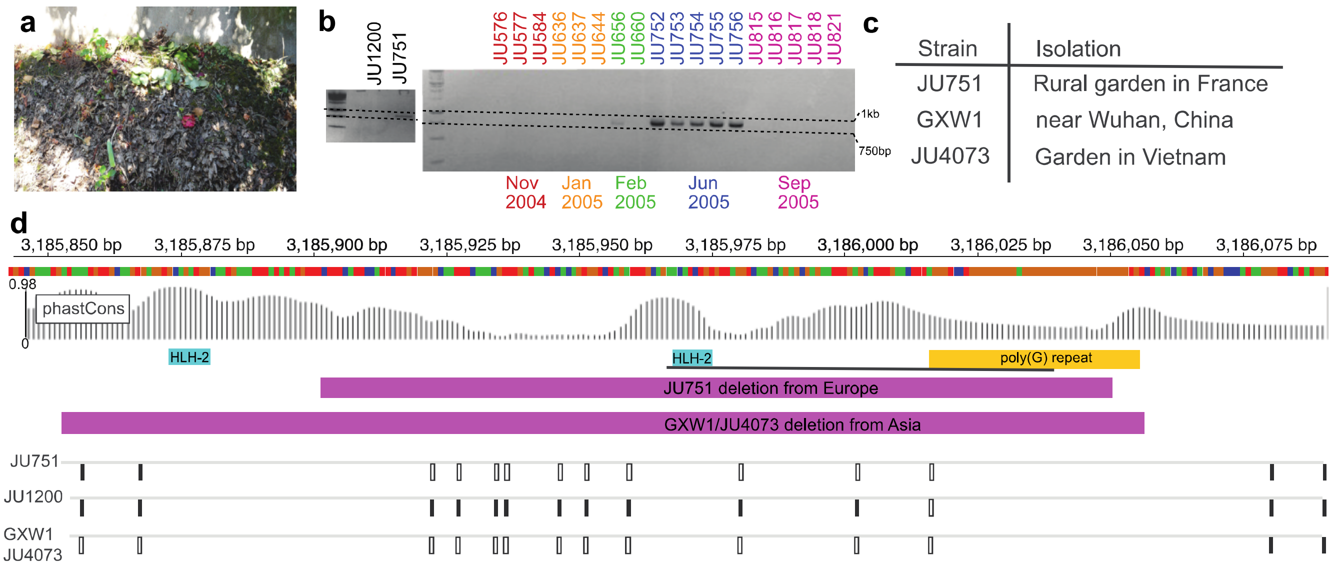

Supplementary Figure 3. The JU751 deletion arose in the wild and is a recent, unique event. a A photograph of the compost pile in Le Perreux-sur-Marne, France from which JU751 and the strains in panel b were isolated[75]. (Picture taken in 2018, Photo credit: Jean-Antoine Lepesant, Institut Jacques Monod, Paris, France). **b** Strains isolated from the compost pile in panel A during the months shown were genotyped for the presence of the *lag-2(cgb1007)* deletion. PCR results from a set of primers spanning the 148 bp deletion are shown. Single PCR across the long polyG repeat is not successful leading to lack of a band in JU1200 and a band of 860 bp in JU751. Strains JU752-756 isolated at the same time and place also give an 860 bp band while other strains isolated from the same place at different times of the year give no band. There are two separate gels shown. This experiment was repeated once with similar results. **c** Isolation locations for JU751 and strains containing a larger, but similar deletion[70]. **d** Variant annotation data from CeNDR showing the deletion region in JU1200, JU751, GXW1, and JU4073[70,74]. The extents of the deletions are marked by the magenta bars. The HLH-2 binding sites are marked by cyan bars. The polyG repeat is marked by a gold bar. Variants from ~1,500 annotated *C. elegans* strains are shown as dark gray solid bars. NO-CALL variants are show as empty bars. Further confirmation that the deletion is only present in JU751 can be found in the CeNDR database (https://elegansvariation.org)[70]. Reference sequence data is from genome WS276[115].

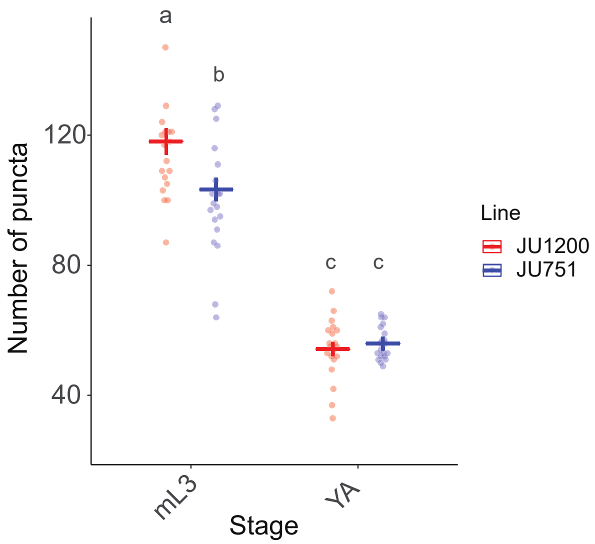

Supplementary Figure 4. *lag-2* expression in the DTC of JU1200 and JU751 at midL4 and young adult.
Data from quantification of *lag-2* transcripts via smFISH at mid-L3 and young adult. Crossbars and error bars represent estimated marginal means +/- standard error from a generalized linear model with a negative binomial distribution. n = 20 for all four groups (single experiment). Lowercase letters indicate significant (p<0.05) Tukey-adjusted pairwise contrasts. For data and statistical results see Supplementary Note 12 and Source Data.

Supplementary Notes

Supplementary Note 1 (accompanies Fig. 1c). Statistical testing for correlation between the number of progenitor zone nuclei and progenitor zone size. Data from two strains (JU1200 and JU751) and two adult stages (L4+24h, YA+24h) were fitted to a gaussian linear model, PZSize_um^2 ~ Cells + Strain + Stage. For data representation (strain and stage aggregated, as their effects are not significant in the model below), see Fig. 1c. Data are provided as a Source Data file.

Correlation between PZ cell number and PZ size: Gaussian linear model

Residual standard error: 321.8 on 83 degrees of freedom
Multiple R-squared 0.7662
Adjusted R-squared 0.758
F-statistic 90.69 on 3 and 83 degrees of freedom
$n = 87$
$p < 2.2E-16$

Source	*Estimate*	*SE*	*t-value*	*Pr>|z|)*	
Intercept	414.842	222.83	1.862	0.0662	
Cells	12.2113	0.9135	13.367	<2.2E-16	***
StrainJU751	115.667	104.17	1.110	0.2700	
StageYA+24	54.7432	82.777	0.661	0.5102	

Supplementary Note 2 (accompanies Fig. 1d). Statistical testing for differences in the size of the progenitor zones among selected wild isolates of *C. elegans* and *C. briggsae*. a Raw data (PZ area in pixels) were fitted to a generalized linear mixed model, PZArea_pixels ~ Strain + (1|Exp), with a negative binomial distribution (log link) and a dispersion parameter of 37.5[106,107]. The R software package, 'emmeans' was used to obtain estimated marginal means on the response scale (**b**) and pairwise contrasts (**c**) with Tukey corrected p-values[108]. **d** An estimate of broad sense heritability (H^2) was derived by calculating the proportion of the variance explained by Strain in an ANOVA (PZArea ~ Strain + Exp) of this same data. Area in pixels was scaled to μm^2/1,000 for representation in Fig. 1d. Data are provided as a Source Data file.

a) PZ Size in wild isolates: Negative binomial model

Source	*Estimate*	*SE*	*Z-value*	*Pr>|z|)*	
Intercept	10.41613	0.0272	382.9	< 2e-16	***
CB4856	-0.14976	0.03268	-4.6	4.59E-06	***
CX11262	-0.04457	0.03417	-1.3	0.192034	
HK104	-0.10483	0.03268	-3.2	0.001337	**
JU1200	0.25508	0.02981	8.6	< 2e-16	***
JU1491	0.15314	0.03425	4.5	7.77E-06	***
JU751	0.06414	0.03001	2.1	0.032583	*
JU775	0.08888	0.03411	2.6	0.009177	**
MY16	0.03845	0.03419	1.1	0.260849	
N2	0.10019	0.02993	3.3	0.000817	***

b) PZ Size in wild isolates: Emmeans

Strain	*n*	*response*	*SE*	*df*
AF16	50	33393.7851	908.415431	1139
CB4856	50	28749.1438	782.118305	1139
CX11262	121	31938.0063	659.990888	1139
HK104	50	30070.3221	818.044304	1139
JU1200	180	43096.8579	707.047176	1139
JU1491	118	38919.9543	809.491218	1139
JU751	169	35605.6964	595.209483	1139
JU775	123	36497.6877	751.01212	1139
MY16	120	34702.6497	718.646237	1139
N2	170	36912.7142	614.851536	1139

c) PZ Size in wild isolates: Contrasts

Contrast	*ratio*	*SE*	*df*	*t-ratio*	*p -value*	
AF16 / CB4856	1.16155755	0.03795836	1139	4.58283737	0.00021846	***
AF16 / CX11262	1.04558139	0.03572386	1139	1.30458463	0.95269564	
AF16 / HK104	1.11052303	0.0362901	1139	3.20796461	0.04458804	*
AF16 / JU1200	0.77485429	0.02310083	1139	-8.5559722	1.27E-13	***
AF16 / JU1491	0.85801193	0.02938501	1139	-4.4714502	0.00036324	***
AF16 / JU751	0.9378776	0.02814523	1139	-2.1371846	0.50138738	
AF16 / JU775	0.91495619	0.03121234	1139	-2.6053949	0.21612601	
AF16 / MY16	0.96228344	0.03290344	1139	-1.1243861	0.98222963	
AF16 / N2	0.90466892	0.02708016	1139	-3.3469298	0.0288436	*
CB4856 / CX11262	0.90015462	0.03075644	1139	-3.0785783	0.06535972	
CB4856 / HK104	0.95606371	0.03124402	1139	-1.3748753	0.93477085	
CB4856 / JU1200	0.66708213	0.01988891	1139	-13.578571	3.41E-14	***
CB4856 / JU1491	0.73867363	0.02529901	1139	-8.8439648	1.20E-13	***
CB4856 / JU751	0.80743102	0.02423192	1139	-7.127276	8.17E-11	***
CB4856 / JU775	0.78769768	0.02687226	1139	-6.9952016	2.03E-10	***
CB4856 / MY16	0.82844233	0.02832821	1139	-5.5040371	2.04E-06	***
CB4856 / N2	0.77884123	0.02331494	1139	-8.3495758	1.48E-13	***
CX11262 / HK104	1.06211055	0.03628968	1139	1.76360516	0.75804118	
CX11262 / JU1200	0.74107505	0.01489957	1139	-14.904163	3.41E-14	***
CX11262 / JU1491	0.8206075	0.0173465	1139	-9.3530432	1.29E-13	***
CX11262 / JU751	0.89699148	0.01823029	1139	-5.3488432	4.75E-06	***
CX11262 / JU775	0.87506931	0.01830673	1139	-6.3790696	1.16E-08	***
CX11262 / MY16	0.92033336	0.01937291	1139	-3.9439331	0.00338054	**
CX11262 / N2	0.8652305	0.01759653	1139	-7.1178928	8.72E-11	***
HK104 / JU1200	0.69773815	0.02080256	1139	-12.07178	3.41E-14	***
HK104 / JU1491	0.77261966	0.0264613	1139	-7.5321861	4.66E-12	***
HK104 / JU751	0.84453683	0.02534509	1139	-5.6302347	1.01E-06	***
HK104 / JU775	0.82389663	0.02810683	1139	-5.6782355	7.71E-07	***
HK104 / MY16	0.86651372	0.02962966	1139	-4.1901175	0.00123886	**

HK104 / N2	0.81463319	0.02438598	1139	-6.8487686	5.47E-10	***
JU1200 / JU1491	1.10732036	0.02241568	1139	5.03591895	2.43E-05	***
JU1200 / JU751	1.21039222	0.02118117	1139	10.9114687	1.09E-13	***
JU1200 / JU775	1.18081064	0.02363407	1139	8.30378008	1.56E-13	***
JU1200 / MY16	1.24188955	0.0250247	1139	10.7508008	1.19E-13	***
JU1200 / N2	1.16753425	0.02039989	1139	8.86495347	1.19E-13	***
JU1491 / JU751	1.09308224	0.02236466	1139	4.3499841	0.00062327	***
JU1491 / JU775	1.06636767	0.02245004	1139	3.0522364	0.07045444	
JU1491 / MY16	1.12152688	0.0237557	1139	5.41466278	3.33E-06	***
JU1491 / N2	1.05437802	0.02158697	1139	2.58630107	0.22521825	
JU751 / JU775	0.97556033	0.01974004	1139	-1.2228218	0.96874758	
JU751 / MY16	1.02602242	0.02089853	1139	1.26124197	0.96180811	
JU751 / N2	0.96459166	0.01711941	1139	-2.0312575	0.57694113	
JU775 / MY16	1.05172625	0.0220481	1139	2.40572088	0.32320127	
JU775 / N2	0.98875654	0.02002054	1139	-0.5584271	0.99992588	
MY16 / N2	0.94012728	0.0191617	1139	-3.0291396	0.07518765	

d) PZ Size in wild isolates: ANOVA to calculate broad sense heritability

	Df	*Sum Sq*	*Mean Sq*	*F value*	*Pr(>F)*
Strain	9	16263025615	1807002846	49.086242	3.69E-75
Exp	1	316046988.4	316046988	8.58524323	0.00345644
Residuals	1141	42003424249	36812817	NA	NA

$H^2 = 16263025615 / (42003424249 + 316046988.4 + 16263025615) = 0.28$

Supplementary Note 3 (accompanies Fig. 2a). Statistical testing for differences in the number of progenitor zone nuclei between JU1200 and JU751 at L4 and adult stages. Data were fitted to a generalized linear model with negative binomial distribution: Cells ~ Strain * Stage, with a dispersion formula ~ Strain * Stage, and log link (**a**, **b**)[106,107]. The R software package, 'emmeans' was used to obtain estimated marginal means on the response scale (**c**) and pairwise contrasts (**d**) with Tukey corrected p-values[108]. For data representation, see Fig. 2a. Data are provided as a Source Data file.

a) PZ cells in JU1200 and JU751: Negative binomial conditional model

Source	*Estimate*	*SE*	*Z-value*	*Pr>|z|)*	
Intercept	5.213	0.0585	89.11	<2e-16	***
StrainJU751	-0.038	0.0866	-0.44	0.6573	
StagemL4+24	0.3053	0.0661	4.62	3.85e-6	***
StageYA	0.4014	0.0637	6.30	2.9e-10	***
StagemYA+24	0.0737	0.0855	0.87	0.3821	
StrainJU751:StagemL4+24	-0.2628	0.1170	-2.25	0.0248	*
StrainJU751:StageYA	-0.0850	0.0945	-0.90	0.3686	
StrainJU751:StageYA+24	0.2221	0.1294	-1.72	0.0862	

b) PZ cells in JU1200 and JU751: Negative binomial dispersion model

Source	*Estimate*	*SE*	*Z-value*	*Pr>|z|)*	
Intercept	2.7645	0.3425	8.072	6.89e-16	***
StrainJU751	-0.2384	0.4751	-0.502	0.6159	
StagemL4+24	1.6540	0.5717	2.893	0.0038	**
StageYA	1.9473	0.5614	3.468	0.0005	***
StagemYA+24	-0.0864	0.4859	-0.178	0.8589	
StrainJU751:StagemL4+24	-1.7624	0.7473	-2.358	0.0184	*
StrainJU751:StageYA	-0.0974	0.7689	-0.127	0.8992	
StrainJU751:StageYA+24	-0.1461	0.6779	-0.215	0.8294	

c) PZ cells in JU1200 and JU751: Emmeans

Strain	*Stage*	*n*	*response*	*SE*	*df*
JU1200	*mL4*	20	183.686	10.427	140
JU751	*mL4*	21	176.762	11.287	140
JU1200	*mL4+24*	17	249.263	7.662	140
JU751	*mL4+24*	18	184.441	13.367	140
JU1200	*YA*	20	274.398	6.896	140
JU751	*YA*	21	242.551	6.840	140
JU1200	*YA+24*	19	197.932	12.331	140
JU751	*YA+24*	20	152.540	11.181	140

d) PZ cells in JU1200 and JU751: Contrasts

Contrast	*ratio*	*SE*	*df*	*t-ratio*	*p -value*	
JU1200 mL4 / JU751 mL4	1.039	0.090	140	0.4437	9.998E-01	
JU1200 mL4 / JU1200 mL4+24	0.737	0.049	140	-4.6194	2.281E-04	***
JU1200 mL4 / JU751 mL4+24	0.996	0.093	140	-0.0440	1.000E+00	
JU1200 mL4 / JU1200 YA	0.669	0.043	140	-6.3033	9.882E-08	***
JU1200 mL4 / JU751 YA	0.757	0.049	140	-4.2803	8.825E-04	***
JU1200 mL4 / JU1200 YA+24	0.928	0.079	140	-0.8740	9.879E-01	
JU1200 mL4 / JU751 YA+24	1.204	0.113	140	1.9812	4.982E-01	
JU751 mL4 / JU1200 mL4+24	0.709	0.050	140	-4.8499	8.691E-05	***
JU751 mL4 / JU751 mL4+24	0.958	0.093	140	-0.4403	9.998E-01	
JU751 mL4 / JU1200 YA	0.644	0.044	140	-6.4087	5.812E-08	***
JU751 mL4 / JU751 YA	0.729	0.051	140	-4.5328	3.247E-04	***
JU751 mL4 / JU1200 YA+24	0.893	0.080	140	-1.2680	9.090E-01	
JU751 mL4 / JU751 YA+24	1.159	0.113	140	1.5161	7.976E-01	
JU1200 mL4+24 / JU751 mL4+24	1.351	0.106	140	3.8258	4.721E-03	**
JU1200 mL4+24 / JU1200 YA	0.908	0.036	140	-2.4195	2.403E-01	
JU1200 mL4+24 / JU751 YA	1.028	0.043	140	0.6544	9.980E-01	
JU1200 mL4+24 / JU1200 YA+24	1.259	0.087	140	3.3192	2.477E-02	*
JU1200 mL4+24 / JU751 YA+24	1.634	0.130	140	6.1786	1.842E-07	***
JU751 mL4+24 / JU1200 YA	0.672	0.052	140	-5.1787	2.072E-05	***
JU751 mL4+24 / JU751 YA	0.760	0.059	140	-3.5218	1.314E-02	*
JU751 mL4+24 / JU1200 YA+24	0.932	0.089	140	-0.7387	9.956E-01	
JU751 mL4+24 / JU751 YA+24	1.209	0.125	140	1.8423	5.925E-01	
JU1200 YA / JU751 YA	1.131	0.043	140	3.2661	2.906E-02	*
JU1200 YA / JU1200 YA+24	1.386	0.093	140	4.8626	8.235E-05	***

JU1200 YA / JU751 YA+24	1.799	0.139	140	7.5776	1.223E-10	***
JU751 YA / JU1200 YA+24	1.225	0.084	140	2.9727	6.643E-02	
JU751 YA / JU751 YA+24	1.590	0.125	140	5.9056	7.024E-07	***
JU1200 YA+24 / JU751 YA+24	1.298	0.125	140	2.7080	1.289E-01	

Supplementary Note 4 (accompanies Fig. 2b). Statistical testing for differences in the number of dividing progenitor zone nuclei (EdU+, 15-minute pulse) between JU1200 and JU751 at the mid L4 stage. Data were fitted to a linear model with gaussian distribution: EdU_Cells ~ Strain with a dispersion estimate (σ^2) of 147 (**a**)[106, 107]. The R software package, 'emmeans' was used to obtain estimated marginal means (**b**) and pairwise contrasts (**c**) with Tukey corrected p-values[108]. For data representation, see Fig. 2b. Data are provided as a Source Data file.

a) EdU+ PZ cells in JU1200 and JU751 at mL4: Gaussian conditional model

Source	*Estimate*	*SE*	*Z-value*	*Pr>|z|)*	
Intercept	100.636	2.585	38.93	<2e-16	***
StrainJU751	-15.956	3.545	-4.50	6.75e-6	***

b) EdU+ PZ cells at mL4 in JU1200 and JU751 at mL4: Emmeans

Strain	*n*	*response*	*SE*	*df*
JU1200	22	100.636	2.585	44
JU751	25	84.680	2.425	44

c) EdU+ PZ cells at mL4 in JU1200 and JU751 at mL4: Contrasts

Contrast	*ratio*	*SE*	*df*	*t-ratio*	*p -value*	
JU1200 / JU751	15.956	3.5447	44	4.502	4.90e-5	***

Supplementary Note 5 (accompanies Fig. 3a). Correction of block effect in the RIL data set. RIL data were collected in six blocks (SB3-SB8), each of which contained both RIL parents. **a** We performed a two-way ANOVA on the parental data alone to determine whether there was an effect of block on PZ Area. Formula: PZArea_pixels ~ Line * Block. **b** Least squares means were used to derive a correction factor for each block by dividing the least squares mean of each block (SB4-SB8) by that of block SB3. Raw PZ area in pixels for each sample was multiplied by the correction factor to generate Corr_PZArea_pixels. The mean corrected PZ area (Corr_PZArea_pixels) for each RIL was used as the phenotype for QTL mapping (Source Data: Fig3a_mapping). Area in pixels was scaled to $\mu m^2/1,000$ for presentation in Figs. 3a, e, and h (0.1008 μm^2/pixel). Data are provided as a Source Data file.

a) ANOVA from only parental data from each block.

	df	*Sum Sq*	*Mean Sq*	*F-value*	*Pr(>F)*	
Line	1	3987203584	3987203584	156.81	5.92E-30	***
Block	5	3376974720	675394944	26.563	9.65E-23	***
Line:Block	5	140124205	28024841	1.1022	0.3589	ns
Residuals	348	8848410248	25426466.23	NA	NA	

b) Calculation of least squares means and correction factors.

Block	*n*	*lsmean*	*SE*	*df*	*lower.CL*	*upper.CL*	*Corr_factor*
SB3	300	33784.6	650.979598	348	32504.2506	35064.9494	1
SB4	510	34671.3167	650.979598	348	33390.9672	35951.6661	1.02624618
SB5	540	36650.4667	650.979598	348	35370.1172	37930.8161	1.0848276
SB6	420	37485.3833	650.979598	348	36205.0339	38765.7328	1.10954054
SB7	540	35956.65	650.979598	348	34676.3006	37236.9994	1.06429113
SB8	510	43251.5333	650.979598	348	41971.1839	44531.8828	1.28021446

Supplementary Note 6 (accompanies Fig. 3j) Statistical data for the multi-QTL model. The fitqtl() function in R/qtl was used to generate model statistics (two-sided) for Corr_PZArea_pixels ~ chrII@pos37.0 + chrV@pos0.0[72].

a) Full model result:
Method: Haley-Knott regression
Model: normal phenotype
Number of observations: 70

	df	*SS*	*MS*	*LOD*	*%var*	*P value (Chi²)*	*P value (F)*	
Model	2	2.0e8	1.0e8	5.85	31.96	1.4e-06	2.5e-06	***
Error	67	4.3e8	6.4e6					
Total	69	6.3e7						

b) Drop one QTL at a time ANOVA table:

	df	*Type III SS*	*LOD*	*%var*	*F value*	*P value (Chi²)*	*P value (F)*	
II@37.0	1	1.3e8	4.036	20.69	20.38	0	2.64e-05	***
V@0.0	1	8.6e7	2.794	13.73	13.52	0	0.000472	***

c) Estimated effects:

	estimate	*SE*	*t*
Intercept	34477.6	303.9	113.437
II@37.0	-1371.9	303.9	-4.514
V@0.0	-1119.6	304.5	-3.677

Supplementary Note 7 (accompanies Fig. 4c). Statistical testing for differences in young adult progenitor zone size among the parental lines and JU1200 NILs. a Data from nine experimental blocks were analyzed together. Raw PZ Area in pixels was fitted to a generalized linear mixed model with a negative binomial distribution and a log link, PZArea_pixels ~ Genotype * (1|Exp) with dispersion formula ~ Genotype[106, 107]. **b, c** The R software package, 'emmeans' was used to obtain estimated marginal means on the response scale and pairwise contrasts with Tukey corrected p-values[108]. Area in pixels was scaled to μm^2/1,000 for presentation in Fig. 4c (0.0504 μm^2/pixel). Data are provided as a Source Data file.

a) PZ size across 9 experiments: Negative binomial conditional models

Random effects:

Groups	*Source*	*Variance*	*Std.Dev*
Exp	Intercept	0.003648	0.0604

Number of obs: 1,557, groups: Exp, 9

Main effects:

Source	*Estimate*	*SE*	*Z-value*	*Pr>|z|)*	
Intercept	11.45866	0.02123	539.7	< 2e-16	***
GenotypeJU751	-0.22330	0.00979	-22.8	< 2e-16	***
GenotypeNIC1697	-0.05175	0.01054	-4.9	9.04e-07	***
GenotypeNIC1701	-0.04231	0.01057	-4.0	6.23e-05	***

Dispersion model:

Source	*Estimate*	*SE*	*Z-value*	*Pr>|z|)*	
Intercept	3.87949	0.06531	59.40	< 2e-16	***
GenotypeJU751	-0.19045	0.09405	-2.02	0.0429	*
GenotypeNIC1697	0.16493	0.10868	1.52	0.1291	
GenotypeNIC1701	0.12415	0.10449	1.19	0.2348	

b) PZ size in JU1200 NILs across 9 experiments: Emmeans

Genotype	*n*	*response*	*SE*	*df*
JU1200	481	94717.8457	2010.82067	1548
JU751	474	75762.3242	1625.40589	1548
NIC1697	289	89940.653	1953.82761	1548
NIC1701	312	90793.8034	1977.71553	1548

c) PZ size in JU1200 NILs across 9 experiments: Contrasts

Contrast	*Ratio*	*SE*	*df*	*t-ratio*	*p -value*	
JU1200 / JU751	1.2501972	0.01223992	1548	22.8082032	<2e-16	***
JU1200 / NIC1697	1.05311494	0.01109685	1548	4.91141477	5.96E-06	***
JU1200 / NIC1701	1.04321927	0.01102459	1548	4.00378144	0.00037965	***
JU751 / NIC1697	0.84235906	0.00922837	1548	-15.65887	<2e-16	***
JU751 / NIC1701	0.83444378	0.00922435	1548	-16.372524	0=<2e-16	***
NIC1697 / NIC1701	0.99060343	0.01155421	1548	-0.8094267	0.85003654	

Supplementary Note 8 (accompanies Fig. 4d). Statistical testing for differences in young adult Progenitor Zone size among the parental lines and JU751 NILs. a Raw data (PZ area in pixels) from one representative experiment of nine were fitted to a generalized linear model with a negative binomial distribution and a log link, PZArea_pixels ~ Strain, with dispersion parameter of 60.2[106, 107]. **b, c** The R software package, 'emmeans' was used to obtain estimated marginal means on the response scale and pairwise contrasts with Tukey corrected p-values[108]. Area in pixels was scaled to μm^2/1,000 for presentation in Fig. 4d (0.1008 μm^2/pixel). Data are provided as a Source Data file.

a) PZ size in parental lines and JU751 NILs: Gaussian conditional model

Source	*Estimate*	*SE*	*Z-value*	*Pr>|z|)*	
Intercept	10.81931	0.01922	562.8	< 2e-16	***
StrainJU751	-0.21814	0.02719	-8	1.03E-15	***
StrainNIC1671	-0.11557	0.02719	-4.3	2.13E-05	***
StrainNIC1672	-0.07027	0.02719	-2.6	0.00974	**
StrainNIC1673	-0.12449	0.02719	-4.6	4.67E-06	***
StrainNIC1675	-0.0306	0.02719	-1.1	0.26036	
StrainNIC1676	-0.2371	0.02719	-8.7	< 2e-16	***

b) PZ size in parental lines and JU751 NILs: Emmeans

Strain	*n*	*response*	*SE*	*df*
JU1200	45	49976.6843	960.742959	307
JU751	45	40181.811	772.561924	307
NIC1671	45	44522.2362	855.950461	307
NIC1672	45	46585.1892	895.583778	307
NIC1673	45	44126.8179	848.353619	307
NIC1675	45	48470.5764	931.807047	307
NIC1676	45	39427.0885	758.061678	307

c) PZ size in parental lines and JU751 NILs: Contrasts

Contrast	*ratio*	*SE*	*df*	*t-ratio*	*p -value*	
JU1200 / JU751	1.24376386	0.03381619	307	8.02329592	1.63E-12	***
JU1200 / NIC1671	1.12251065	0.03051836	307	4.2507566	0.00055904	***
JU1200 / NIC1672	1.072802	0.02916645	307	2.58481887	0.13424479	
JU1200 / NIC1673	1.13256942	0.03079193	307	4.57887184	0.00013742	***
JU1200 / NIC1675	1.03107262	0.02803161	307	1.12553123	0.91991339	
JU1200 / NIC1676	1.26757228	0.03446375	307	8.72063522	1.17E-12	***
JU751 / NIC1671	0.90251107	0.02453891	307	-3.7725581	0.00362754	**
JU751 / NIC1672	0.86254476	0.02345188	307	-5.4384959	2.28E-06	***
JU751 / NIC1673	0.91059843	0.02475888	307	-3.4444426	0.01150048	*
JU751 / NIC1675	0.82899388	0.02253939	307	-6.8977734	6.35E-10	***
JU751 / NIC1676	1.01914223	0.02771128	307	0.69734372	0.99268438	
NIC1671 / NIC1672	0.95571655	0.02598419	307	-1.6659417	0.63930687	
NIC1671 / NIC1673	1.00896095	0.02743231	307	0.32811614	0.9998988	
NIC1671 / NIC1675	0.91854151	0.02497316	307	-3.1252274	0.03167016	*
NIC1671 / NIC1676	1.12922962	0.03070351	307	4.46990136	0.00022128	***
NIC1672 / NIC1673	1.0557115	0.02870295	307	1.99405778	0.42054372	
NIC1672 / NIC1675	0.96110244	0.0261299	307	-1.4592878	0.76863618	
NIC1672 / NIC1676	1.18155286	0.03212567	307	6.13583829	5.46E-08	***
NIC1673 / NIC1675	0.9103836	0.02475144	307	-3.453343	0.01116249	*
NIC1673 / NIC1676	1.11920052	0.03043091	307	4.14178602	0.0008723	***
NIC1675 / NIC1676	1.22937245	0.03342545	307	7.59511412	9.00E-12	***

Supplementary Note 9 (accompanies Fig. 5b). Statistical testing for differences in young adult Progenitor Zone size among the parents and ARLs. a Raw data (PZ area in pixels) were fitted to a generalized linear model with a negative binomial distribution and log link, PZArea_pixels ~ Strain, with a dispersion parameter of 51.4[106, 107]. **b, c** The R software package, 'emmeans' was used to obtain estimated marginal means on the response scale and pairwise contrasts with Tukey corrected p-values[108]. Area in pixels was scaled to μm^2/1,000 for presentation in Fig. 5b (0.0504 μm^2/pixel). Data are provided as a Source Data file.

a) PZ size in parental lines and ARLs: Negative binomial conditional model

Source	*Estimate*	*SE*	*Z-value*	*Pr>|z|)*	
Intercept	11.51157	0.01179	976.3	<2e-16	***
StrainJU751	-0.18316	0.01680	-10.9	<2e-16	***
StrainNIC1720	-0.18556	0.01680	-11.0	<2e-16	***
StrainNIC1725	-0.27452	0.01674	-16.4	<2e-16	***

b) PZ size in parental lines and ARLs: Emmeans

Strain	*n*	*response*	*SE*	*df*
JU1200	140	99854.4059	1177.32195	545
JU751	136	83142.2612	994.641831	545
NIC1720	136	82943.0346	992.260739	545
NIC1725	138	75883.0906	901.224303	545

c) PZ size in parental lines and ARLs: Contrasts

Contrast	*ratio*	*SE*	*df*	*t-ratio*	*p -value*	
JU1200 / JU751	1.20100662	0.02017297	545	10.9045148	3.89E-10	***
JU1200 / NIC1720	1.2038914	0.02022145	545	11.0473327	3.89E-10	***
JU1200 / NIC1725	1.31589798	0.0220217	545	16.4037903	3.89E-10	***
JU751 / NIC1720	1.00240197	0.01695908	545	0.14180313	0.99898218	
JU751 / NIC1725	1.09566256	0.01846987	545	5.41957756	5.37E-07	***
NIC1720 / NIC1725	1.09303712	0.01842564	545	5.27725369	1.13E-06	***

Supplementary Note 10 (accompanies Fig. 5d-f). Statistical testing for differences in DTC *lag-2* expression (smFISH) among the parents and ARLs. **a** Data were fitted to a generalized linear model with a negative binomial distribution and log link, Puncta ~ Strain * Stage, with a dispersion parameter of 24.7[106, 107]. **b, c** The R software package, 'emmeans' was used to obtain estimated marginal means on the response scale and pairwise contrasts with Tukey corrected p-values[108]. For data representation, see Fig 5d-f. Data are provided as a Source Data file.

a) *lag-2* expression in parental lines and ARLs: Negative binomial conditional model

Source	*Estimate*	*SE*	*Z-value*	*Pr>|z|)*	
Intercept	4.130	0.053	77.59	<2e-16	***
StrainJU751	-0.2347	0.077	-3.06	0.0022	**
StrainNIC1720	-0.0721	0.075	0.96	0.3354	
StrainNIC1725	-0.4986	0.079	-6.34	2.31E-10	***
StageeL3	0.6343	0.073	8.72	<2e-16	***
StagemL4	0.0563	0.075	0.75	0.4526	
StrainJU751:StageeL3	-0.0140	0.104	-0.13	0.8937	
StrainNIC1720:StageeL3	-0.6532	0.104	-6.27	3.6E-10	***
StrainNIC1725:StageeL3	-0.0016	0.107	0.01	0.9882	
StrainJU751:StagemL4	0.3358	0.107	3.15	0.0016	**
StrainNIC1720:StagemL4	-0.0906	0.106	-0.86	0.3921	
StrainNIC1725:StagemL4	0.4617	0.109	4.23	2.39E-05	***

b) *lag-2* expression in parental lines and ARLs: Emmeans

Strain	*Stage*	*n*	*response*	*SE*	*df*
JU1200	*mL2*	20	62.150	3.308	226
JU751	*mL2*	20	49.150	2.712	226
NIC1720	*mL2*	20	66.800	3.520	226
NIC1725	*mL2*	20	37.750	2.186	226
JU1200	*eL3*	20	117.20	5.807	226
JU751	*eL3*	20	91.400	4.638	226
NIC1720	*eL3*	20	65.550	3.463	226
NIC1725	*eL3*	20	71.300	3.725	226
JU1200	*mL4*	20	65.750	3.472	226
JU751	*mL4*	20	72.750	3.791	226
NIC1720	*mL4*	20	64.550	3.417	226
NIC1725	*mL4*	19	63.368	3.451	226

c) *lag-2* expression in parental lines and ARLs: Contrasts

Contrast	*ratio*	*SE*	*df*	*t-ratio*	*p -value*	
JU1200 mL2 / JU751 mL2	1.264	0.097	226	3.061	0.0985	
JU1200 mL2 / NIC1720 mL2	0.9303	0.070	226	-0.9633	0.9983	
JU1200 mL2 / NIC1725 mL2	1.6464	0.129	226	6.339	8.13E-08	***
JU1200 mL2 / JU1200 eL3	0.5303	0.039	226	-8.723	1.50E-13	***
JU1200 mL2 / JU751 eL3	0.6800	0.050	226	-5.245	2.30E-05	***
JU1200 mL2 / NIC1720 eL3	0.9481	0.071	226	-0.7102	0.9999	
JU1200 mL2 / NIC1725 eL3	0.8717	0.065	226	-1.842	0.7932	
JU1200 mL2 / JU1200 mL4	0.9452	0.071	226	-0.7510	0.9998	
JU1200 mL2 / JU751 mL4	0.8543	0.064	226	-2.114	0.6132	
JU1200 mL2 / NIC1720 mL4	0.9628	0.072	226	-0.5047	>0.99999	
JU1200 mL2 / NIC1725 mL4	0.9808	0.075	226	-0.2550	>0.99999	
JU751 mL2 / NIC1720 mL2	0.7358	0.056	226	-4.021	0.0044	**
JU751 mL2 / NIC1725 mL2	1.302	0.104	226	3.299	0.0505	
JU751 mL2 / JU1200 eL3	0.4194	0.031	226	-11.717	1.41E-14	***
JU751 mL2 / JU751 eL3	0.5377	0.040	226	-8.275	8.58E-13	***
JU751 mL2 / NIC1720 eL3	0.7498	0.057	226	-3.769	0.0111	*
JU751 mL2 / NIC1725 eL3	0.6893	0.052	226	-4.895	0.0001	***
JU751 mL2 / JU1200 mL4	0.7475	0.057	226	-3.810	0.0096	**
JU751 mL2 / JU751 mL4	0.6756	0.051	226	-5.167	3.33E-05	***
JU751 mL2 / NIC1720 mL4	0.7614	0.058	226	-3.564	0.0221	*
JU751 mL2 / NIC1725 mL4	0.7756	0.060	226	-3.277	0.0539	
NIC1720 mL2 / NIC1725 mL2	1.770	0.139	226	7.290	3.41E-10	***
NIC1720 mL2 / JU1200 eL3	0.5700	0.041	226	-7.772	1.78E-11	***
NIC1720 mL2 / JU751 eL3	0.7308	0.053	226	-4.286	0.0016	**
NIC1720 mL2 / NIC1720 eL3	1.019	0.076	226	0.2532	>0.99999	
NIC1720 mL2 / NIC1725 eL3	0.9370	0.070	226	-0.8786	0.9993	
NIC1720 mL2 / JU1200 mL4	1.016	0.076	226	0.2123	>0.99999	

NIC1720 mL2 / JU751 mL4	0.9182	0.068	226	-1.151	0.9918	
NIC1720 mL2 / NIC1720 mL4	1.035	0.077	226	0.4587	>0.99999	
NIC1720 mL2 / NIC1725 mL4	1.054	0.080	226	0.6959	>0.99999	
NIC1725 mL2 / JU1200 eL3	0.3221	0.025	226	-14.866	<2e-16	***
NIC1725 mL2 / JU751 eL3	0.4130	0.032	226	-11.485	2.78E-14	***
NIC1725 mL2 / NIC1720 eL3	0.5759	0.045	226	-7.040	1.50E-09	***
NIC1725 mL2 / NIC1725 eL3	0.5295	0.041	226	-8.154	1.72E-12	***
NIC1725 mL2 / JU1200 mL4	0.5741	0.045	226	-7.081	1.18E-09	***
NIC1725 mL2 / JU751 mL4	0.5189	0.040	226	-8.422	3.97E-13	***
NIC1725 mL2 / NIC1720 mL4	0.5848	0.046	226	-6.837	4.89E-09	***
NIC1725 mL2 / NIC1725 mL4	0.5957	0.047	226	-6.517	3.04E-08	***
JU1200 eL3 / JU751 eL3	1.282	0.091	226	3.506	0.0267	*
JU1200 eL3 / NIC1720 eL3	1.788	0.129	226	8.023	3.80E-12	***
JU1200 eL3 / NIC1725 eL3	1.644	0.118	226	6.902	3.36E-09	***
JU1200 eL3 / JU1200 mL4	1.783	0.129	226	7.982	4.87E-12	***
JU1200 eL3 / JU751 mL4	1.611	0.116	226	6.632	1.59E-08	***
JU1200 eL3 / NIC1720 mL4	1.816	0.132	226	8.226	1.12E-12	***
JU1200 eL3 / NIC1725 mL4	1.850	0.136	226	8.352	5.65E-13	***
JU751 eL3 / NIC1720 eL3	1.394	0.102	226	4.538	0.0006	***
JU751 eL3 / NIC1725 eL3	1.282	0.093	226	3.410	0.0361	*
JU751 eL3 / JU1200 mL4	1.390	0.102	226	4.497	0.0007	***
JU751 eL3 / JU751 mL4	1.256	0.091	226	3.138	0.0801	
JU751 eL3 / NIC1720 mL4	1.416	0.104	226	4.743	0.0002	***
JU751 eL3 / NIC1725 mL4	1.442	0.107	226	4.921	0.0001	***
NIC1720 eL3 / NIC1725 eL3	0.9194	0.068	226	-1.132	0.9929	
NIC1720 eL3 / JU1200 mL4	0.9970	0.074	226	-0.041	>0.99999	
NIC1720 eL3 / JU751 mL4	0.9010	0.067	226	-1.404	0.9617	
NIC1720 eL3 / NIC1720 mL4	1.015	0.076	226	0.206	>0.99999	
NIC1720 eL3 / NIC1725 mL4	1.034	0.078	226	0.446	>0.99999	
NIC1725 eL3 / JU1200 mL4	1.084	0.081	226	1.091	0.9948	
NIC1725 eL3 / JU751 mL4	0.9801	0.072	226	-0.273	>0.99999	
NIC1725 eL3 / NIC1720 mL4	1.105	0.082	226	1.337	0.9733	
NIC1725 eL3 / NIC1725 mL4	1.125	0.085	226	1.563	0.9205	
JU1200 mL4 / JU751 mL4	0.9038	0.067	226	-1.364	0.9691	
JU1200 mL4 / NIC1720 mL4	1.019	0.076	226	0.2463	>0.99999	
JU1200 mL4 / NIC1725 mL4	1.038	0.079	226	0.4864	>0.99999	
JU751 mL4 / NIC1720 mL4	1.127	0.084	226	1.610	0.9039	
JU751 mL4 / NIC1725 mL4	1.148	0.087	226	1.832	0.7988	
NIC1720 mL4 / NIC1725 mL4	1.019	0.077	226	0.2433	>0.99999	

Supplementary Note 11 accompanies Fig. 6. Statistical testing for differences in PZ area among the parents, NILs, and ARLs (Interaction Data Set) due to the genotypes of the background (BGND) the central portion of the chromosome II QTL (C2) and the chromosome V *lag-2* variant (C5). **a** Raw data (PZ area in pixels) were fitted to a generalized linear model with a negative binomial distribution and log link, PZArea_pixels ~ Background * C2 * C5 + Block + Block:Background + Block:C2 with a dispersion parameter of 44.55[106, 107]. Experimental block was included as fixed effect term with significant interactions in the model. Model selection was conducted using an 'up-down' approach to optimize both the Baysian Information Criterion (BIC) and the adjusted deviance accounted for in the model (D^2) using the R software package, modEvA[78]. The package, 'emmeans' was used to obtain estimated marginal means on the response scale (**b-d**) and pairwise contrasts for the fixed effects (**f**) and interactions (**g**) with Tukey corrected p-values[108]. Analysis was conducted on raw data (PZ area in pixels) and y-axes were scaled to 'PZ Size' in μm^2/1,000 for data representation in Fig. 6 (0.0504 μm^2/pixel). Data are provided as a Source Data file.

Deviance Residuals:

Min	1Q	Med	3Q	Max
-3.748	-0.73	-0.031	0.656	3.033

Null deviance: 4233.7 on 2785 degrees of freedom
Residual deviance: 2796.5 on 2763 degrees of freedom
AIC: 60439
BIC: 60581.48
D^2 = 0.3339762
$P\chi^2$= 0.3237755

a) PZ Area from Interaction Data Set: Negative binomial conditional model

Source	*estimate*	*SE*	*Z-value*	*Pr>|z|)*	
Intercept	11.35674	0.01681	675.679	< 2e-16	***
Background JU751	-0.12907	0.01872	-6.893	5.44E-12	***
C2 JU751	-0.0436	0.0192	-2.27	0.023188	*
C5 JU751	-0.12523	0.01437	-8.712	< 2e-16	***
Block A3	0.18049	0.02117	8.524	< 2e-16	***
Block B1	0.07787	0.02053	3.792	0.000149	***
Block B2	0.13552	0.02021	6.706	1.99E-11	***
Block N3	0.19258	0.02371	8.122	4.57E-16	***
Block N5	0.07279	0.02559	2.845	0.004442	**
Background JU751:C2 JU751	-0.06228	0.01855	-3.357	0.000788	***
Background JU751:C5 JU751	0.15208	0.01932	7.872	3.48E-15	***
C2 JU751:C5 JU751	0.06673	0.01906	3.501	0.000463	***
Background JU751: Block A3	-0.17217	0.02511	-6.857	7.02E-12	***
Background JU751: Block B1	-0.02981	0.02249	-1.326	0.184893	
Background JU751: Block B2	-0.04634	0.02165	-2.14	0.03235	*
Background JU751: Block N3	-0.02818	0.02474	-1.139	0.254816	
Background JU751: Block N5	-0.13209	0.02683	-4.923	8.52E-07	***
C2 JU751: Block A3	-0.06427	0.0235	-2.735	0.006239	**
C2 JU751: Block B1	0.00655	0.02093	0.313	0.754354	
C2 JU751: Block B2	0.04348	0.02016	2.157	0.031009	*
C2 JU751: Block N3	-0.05689	0.02418	-2.353	0.018624	*
C2 JU751: Block N5	-0.02327	0.02613	-0.891	0.373151	
Background JU751:C2 JU751:C5 JU751	-0.01975	0.02669	-0.74	0.459272	

b) PZ Area from Interaction Data Set: Emmeans (Background, C2, C5)

Background	*C2*	*C5*	*n*	*response*	*SE*	*df*
JU1200	*JU1200*	*JU1200*	345	95474.4935	786.532429	Inf
JU751	*JU1200*	*JU1200*	395	78390.0105	701.590905	Inf
JU1200	*JU751*	*JU1200*	314	89974.616	819.559659	Inf
JU751	*JU751*	*JU1200*	286	69413.5523	773.624993	Inf
JU1200	*JU1200*	*JU751*	200	84236.665	1058.236	Inf
JU751	*JU1200*	*JU751*	454	80523.564	647.430365	Inf
JU1200	*JU751*	*JU751*	412	84862.5242	779.008679	Inf
JU751	*JU751*	*JU751*	380	74732.61	589.913664	Inf

c) PZ Area from Interaction Data Set: Emmeans (Background, C2)

Background	*C2*	*n*	*response*	*SE*	*df*
JU1200	*JU1200*	545	89679.7241	701.530732	Inf
JU751	*JU1200*	849	79449.6258	433.220653	Inf
JU1200	*JU751*	726	87381.1938	508.136821	Inf

JU751	*JU751*	666	72023.9955	497.934192	Inf

d) PZ Area from Interaction Data Set: Emmeans (Background, C5)

Background	*C5*	*n*	*response*	*SE*	*df*
JU1200	*JU1200*	659	92683.7682	562.992739	Inf
JU751	*JU1200*	681	73765.3652	551.563165	Inf
JU1200	*JU751*	612	84549.0155	700.082394	Inf
JU751	*JU751*	834	77574.0685	439.703409	Inf

e) PZ Area from Interaction Data Set: Emmeans (C2, C5)

C2	*C5*	*n*	*response*	*SE*	*df*
JU1200	*JU1200*	750	86511.54	527.213468	Inf
JU751	*JU1200*	600	79028.2083	554.518207	Inf
JU1200	*JU751*	654	82359.1919	612.814813	Inf
JU751	*JU751*	792	79636.6619	479.872666	Inf

f) PZ Area from Interaction Data Set: Contrasts

Contrast (Background C2 C5 / Background C2 C5)	*ratio*	*SE*	*df*	*t-ratio*	*p-value*	
JU1200 JU1200 JU1200 / JU751 JU1200 JU1200	1.2179	0.0148	Inf	16.2405	<2e-16	***
JU1200 JU1200 JU1200 / JU1200 JU751 JU1200	1.0611	0.0132	Inf	4.7798	4.80E-05	***
JU1200 JU1200 JU1200 / JU751 JU751 JU1200	1.3754	0.0195	Inf	22.5214	<2e-16	***
JU1200 JU1200 JU1200 / JU1200 JU1200 JU751	1.1334	0.0163	Inf	8.7124	6.54E-14	***
JU1200 JU1200 JU1200 / JU751 JU1200 JU751	1.1857	0.0137	Inf	14.7176	<2e-16	***
JU1200 JU1200 JU1200 / JU1200 JU751 JU751	1.1250	0.0137	Inf	9.6894	9.66E-14	***
JU1200 JU1200 JU1200 / JU751 JU751 JU751	1.2775	0.0147	Inf	21.3024	<2e-16	***
JU751 JU1200 JU1200 / JU1200 JU751 JU1200	0.8712	0.0110	Inf	-10.919	7.77E-14	***
JU751 JU1200 JU1200 / JU751 JU751 JU1200	1.1293	0.0154	Inf	8.9414	5.58E-14	***
JU751 JU1200 JU1200 / JU1200 JU1200 JU751	0.9306	0.0144	Inf	-4.6344	9.72E-05	***
JU751 JU1200 JU1200 / JU751 JU1200 JU751	0.9735	0.0127	Inf	-2.0561	0.4438	
JU751 JU1200 JU1200 / JU1200 JU751 JU751	0.9237	0.0119	Inf	-6.1626	2.00E-08	***
JU751 JU1200 JU1200 / JU751 JU751 JU751	1.0489	0.0126	Inf	3.9874	0.0017	**
JU1200 JU751 JU1200 / JU751 JU751 JU1200	1.2962	0.0191	Inf	17.5948	<2e-16	***
JU1200 JU751 JU1200 / JU1200 JU1200 JU751	1.0681	0.0172	Inf	4.0975	0.0012	**
JU1200 JU751 JU1200 / JU751 JU1200 JU751	1.1174	0.0137	Inf	9.0820	1.04E-13	***
JU1200 JU751 JU1200 / JU1200 JU751 JU751	1.0602	0.0150	Inf	4.1444	0.0009	***
JU1200 JU751 JU1200 / JU751 JU751 JU751	1.2040	0.0145	Inf	15.4204	<2e-16	***
JU751 JU751 JU1200 / JU1200 JU1200 JU751	0.8240	0.0143	Inf	-11.159	2.49E-14	***
JU751 JU751 JU1200 / JU751 JU1200 JU751	0.8620	0.0121	Inf	-10.544	7.37E-14	***
JU751 JU751 JU1200 / JU1200 JU751 JU751	0.8180	0.0116	Inf	-14.157	<2e-16	***
JU751 JU751 JU1200 / JU751 JU751 JU751	0.9288	0.0125	Inf	-5.4750	1.22E-06	***
JU1200 JU1200 JU751 / JU751 JU1200 JU751	1.0461	0.0156	Inf	3.0156	0.0523	.
JU1200 JU1200 JU751 / JU1200 JU751 JU751	0.9926	0.0144	Inf	-0.5109	0.9996	
JU1200 JU1200 JU751 / JU751 JU751 JU751	1.1272	0.0169	Inf	7.9853	1.48E-13	***
JU751 JU1200 JU751 / JU1200 JU751 JU751	0.9489	0.0116	Inf	-4.2780	0.0005	***
JU751 JU1200 JU751 / JU751 JU751 JU751	1.0775	0.0121	Inf	6.6648	7.42E-10	***
JU1200 JU751 JU751 / JU751 JU751 JU751	1.1355	0.0138	Inf	10.4520	7.75E-14	***

g) PZ Area from Interaction Data Set: Contrasts of Interactions

model term	*by*	*df1*	*df2*	*F-ratio*	*p -value*	
Background:C2	*null*	1	Inf	32.599	<0.0001	***
Background:C5	*null*	1	Inf	96.984	<0.0001	***
C2:C5	*null*	1	Inf	15.452	<0.0001	***
Background:C2	*C5=JU1200*	1	Inf	11.269	0.0008	***
Background:C2	*C5=JU751*	1	Inf	20.304	<0.0001	***
Background:C5	*C2=JU1200*	1	Inf	61.967	<0.0001	***
Background:C5	*C2=JU751*	1	Inf	43.773	<0.0001	***
C2:C5	*Background =JU1200*	1	Inf	12.258	0.0005	***
C2:C5	*Background =JU751*	1	Inf	6.877	0.0087	**
Background:C2:C5	*null*	1	Inf	0.548	0.4593	

Supplementary Note 12 accompanies Supplementary Fig. 4. **Statistical testing for differences in DTC *lag-2* expression in JU1200 and JU751 at mid L3 and young adult. a** Data were fitted to a generalized linear model with a negative binomial distribution and log link, Puncta ~ Strain * Stage with a dispersion parameter of 66.4[106, 107]. **b, c** The R software package, 'emmeans' was used to obtain estimated marginal means on the response scale and pairwise contrasts with Tukey corrected p-values[108]. For data representation, see Supplementary Fig. 4. Data are provided as a Source Data file.

a) Number of *lag-2* puncta in parental lines: Negative binomial conditional model

Source	*Estimate*	*SE*	*Z-value*	*Pr>|z|)*	
Intercept	4.77153	0.03429	139.15	<2e-16	***
StrainJU751	-0.13389	0.04912	-2.73	0.00641	**
StageYA	-0.77793	0.05339	-14.57	<2e-16	***
StrainJU751:StageYA	-0.16475	0.07672	2.18	0.02956	*

b) Number of *lag-2* puncta in parental lines: Emmeans

Strain	*Stage*	*n*	*response*	*SE*	*df*
JU1200	*mL3*	20	118.10	4.050	75
JU751	*mL3*	20	103.30	3.633	75
JU1200	*YA*	20	54.25	2.220	75
JU751	*YA*	20	55.95	2.270	75

c) Number of *lag-2* puncta in parental lines: Contrasts

Contrast	*ratio*	*SE*	*df*	*t-ratio*	*p -value*	
JU1200 mL3 / JU751 mL3	1.143	0.056	75	2.726	0.0390	*
JU1200 mL3 / JU1200 YA	2.177	0.116	75	14.572	<2e-16	***
JU1200 mL3 / JU751 YA	2.111	0.112	75	14.063	<2e-16	***
JU751 mL3 / JU1200 YA	1.904	0.103	75	11.937	<2e-16	***
JU751 mL3 / JU751 YA	1.846	0.099	75	11.420	<2e-16	***
JU1200 YA / JU751 YA	0.970	0.056	75	-0.535	0.9501	
