# Supplementary material for: Higher-order epistasis shapes natural variation in germ stem cell niche activity
Source: Nat Commun. 2023 May 17;14:2824. doi: 10.1038/s41467-023-38527-0 (PMC10192456; doi:10.1038/s41467-023-38527-0)
Supplement: Supplementary file 3 — Description of Additional Supplementary Files [file 41467_2023_38527_MOESM3_ESM.pdf]

**Description of Additional Supplementary Files**

File Name: Supplementary Data 1
Description: A table listing all of the oligonucleotides used in this study

File Name: Supplementary Data 2
Description: A table listing all of the strains used in this study
