# Supplementary material for: Higher-order epistasis shapes natural variation in germ stem cell niche activity
Source: Nat Commun. 2023 May 17;14:2824. doi: 10.1038/s41467-023-38527-0 (PMC10192456; doi:10.1038/s41467-023-38527-0)
Supplement: Supplementary file 6 — Reporting Summary [file 41467_2023_38527_MOESM6_ESM.pdf]

nature portfolio

Corresponding author(s): Christian Braendle, Sarah Fausett

Last updated by author(s): Mar 7, 2023

Reporting Summary

Nature Portfolio wishes to improve the reproducibility of the work that we publish. This form provides structure for consistency and transparency in reporting. For further information on Nature Portfolio policies, see our Editorial Policies and the Editorial Policy Checklist.

Statistics

For all statistical analyses, confirm that the following items are present in the figure legend, table legend, main text, or Methods section.

n/a	Confirmed	
☐	☒	The exact sample size (*n*) for each experimental group/condition, given as a discrete number and unit of measurement
☐	☒	A statement on whether measurements were taken from distinct samples or whether the same sample was measured repeatedly
☐	☒	The statistical test(s) used AND whether they are one- or two-sided<br>*Only common tests should be described solely by name; describe more complex techniques in the Methods section.*
☐	☒	A description of all covariates tested
☐	☒	A description of any assumptions or corrections, such as tests of normality and adjustment for multiple comparisons
☐	☒	A full description of the statistical parameters including central tendency (e.g. means) or other basic estimates (e.g. regression coefficient) AND variation (e.g. standard deviation) or associated estimates of uncertainty (e.g. confidence intervals)
☐	☒	For null hypothesis testing, the test statistic (e.g. *F*, *t*, *r*) with confidence intervals, effect sizes, degrees of freedom and *P* value noted<br>*Give P values as exact values whenever suitable.*
☒	☐	For Bayesian analysis, information on the choice of priors and Markov chain Monte Carlo settings
☐	☒	For hierarchical and complex designs, identification of the appropriate level for tests and full reporting of outcomes
☐	☒	Estimates of effect sizes (e.g. Cohen's *d*, Pearson's *r*), indicating how they were calculated

Our web collection on statistics for biologists contains articles on many of the points above.

Software and code

Policy information about availability of computer code

Data collection	Image J2 v2.9.0/1.53t ("Measure" function was used to count pixels--area--in a region of interest to determine PZ size).
Data analysis	Statistical analyses were performed using R Statistical Software (R version 4.2.0, RStudio 2022.02.3 build 492).<br><br>For QTL mapping, PZ size was normalized across blocks using a correction factor. The correction factor was derived from the least square means of each block (lsmeans package v. 2.30-0) to each observation (correction factor = LSMBlockX/LSMBlock1). Normalized PZ size was mapped using the software package R/qtl (v. 1.50).<br><br>For other statistical analyses, R software was used to build generalized linear mixed models.<br><br>Data were fitted to either gaussian or negative binomial (nbinom2) models using the glmmTMB package v. 1.1.3. Residual diagnostics were performed using the DHARMa package v. 0.4.5 according to the developer's guidelines. When model selection was warranted, the Bayesian information criterion (BIC) was used to select the optimal model unless otherwise indicated. The emmeans package v. 1.7.4-1 was used to calculate model estimated marginal means, standard errors, and Tukey-adjusted p-values for pairwise contrasts. For model fitting of the interaction data set corresponding to Figure 6, D-squared was calculated using the modEvA package v. 3.0. Specific details of each model are described in the supplemental table legends, which also display the statistical results (Tables S1-S12). Other R packages used for data manipulation and plotting included tidyverse v. 1.3.1, MASS v. 7.3-57, ggbeeswarm v. 0.6.0, rnaturalearth and rnaturalearthdata v. 0.1.0, rgeos v. 0.5-9 and sf v. 1.0-7

For manuscripts utilizing custom algorithms or software that are central to the research but not yet described in published literature, software must be made available to editors and reviewers. We strongly encourage code deposition in a community repository (e.g. GitHub). See the Nature Portfolio guidelines for submitting code & software for further information.

Data

Policy information about availability of data

All manuscripts must include a data availability statement. This statement should provide the following information, where applicable:

- Accession codes, unique identifiers, or web links for publicly available datasets
- A description of any restrictions on data availability
- For clinical datasets or third party data, please ensure that the statement adheres to our policy

Raw data are available in Data S1. The following publicly available databases were referenced and used in this study: Wormbase (https://wormbase.org/#012-34-5) and the C. elegans Natural Diversity Resource (https://elegansvariation.org/).

Human research participants

Policy information about studies involving human research participants and Sex and Gender in Research.

Reporting on sex and gender	N/A
Population characteristics	N/A
Recruitment	N/A
Ethics oversight	N/A

Note that full information on the approval of the study protocol must also be provided in the manuscript.

Field-specific reporting

Please select the one below that is the best fit for your research. If you are not sure, read the appropriate sections before making your selection.

☒ Life sciences ☐ Behavioural & social sciences ☐ Ecological, evolutionary & environmental sciences

For a reference copy of the document with all sections, see nature.com/documents/nr-reporting-summary-flat.pdf

Life sciences study design

All studies must disclose on these points even when the disclosure is negative.

Sample size	20-40 samples were measured for each independent group. This value was chosen based on a sample size calculator (http://powerandsamplesize.com/Calculators/Compare-k-Means/1-Way-ANOVA-Pairwise). Parameters: 100-400 pairwise comparisons per experimental block; two groups (the parental strains) with means of 47633 and 37584 pixels and 6640 standard deviation in a pilot study; Type I and Type II error rates set to 5%.
Data exclusions	When modeling, influential points were excluded based on a cook's distance cutoff of 6/n.
Replication	Experiments were repeated 1-9 times. Attempts at replication were successful. It should be noted that PZ size is sensitive to environmental conditions including temperature, worm density, and the presence of contamination on the plates. To successfully reproduce the results, steps should be taken to ensure that lines are raised on "clean" uncrowded plates as described in the Materials and Methods and plates are stored in a 20C incubator.
Randomization	N/A
Blinding	Images were given random filenames by manually reassigning names based on a random number sequence before PZ size measurement. A key was created in this process. After measurements, the key was used to match the filenames back up to the identifying information including line or strain name.

Reporting for specific materials, systems and methods

We require information from authors about some types of materials, experimental systems and methods used in many studies. Here, indicate whether each material, system or method listed is relevant to your study. If you are not sure if a list item applies to your research, read the appropriate section before selecting a response.

Materials & experimental systems

n/a	Involved in the study
☒	☐ Antibodies
☒	☐ Eukaryotic cell lines
☒	☐ Palaeontology and archaeology
☐	☒ Animals and other organisms
☒	☐ Clinical data
☒	☐ Dual use research of concern

Methods

n/a	Involved in the study
☒	☐ ChIP-seq
☒	☐ Flow cytometry
☒	☐ MRI-based neuroimaging

Animals and other research organisms

Policy information about studies involving animals; ARRIVE guidelines recommended for reporting animal research, and Sex and Gender in Research

Laboratory animals

AF16, C. briggsae
CB4856 C. elegans
CX11262 C. elegans
HK104 C. briggsae
JU576 C. elegans
JU577 C. elegans
JU584 C. elegans
JU636 C. elegans
JU637 C. elegans
JU644 C. elegans
JU656 C. elegans
JU660 C. elegans
JU751 C. elegans
JU752 C. elegans
JU753 C. elegans
JU754 C. elegans
JU755 C. elegans
JU756 C. elegans
JU775 C. elegans
JU815 C. elegans
JU816 C. elegans
JU817 C. elegans
JU818 C. elegans
JU821 C. elegans
JU1200 C. elegans
JU1491 C. elegans
N2 C. elegans
NIC601 C. elegans
NIC606 C. elegans
NIC610 C. elegans
NIC617 C. elegans
NIC619 C. elegans
NIC621 C. elegans
NIC623 C. elegans
NIC624 C. elegans
NIC625 C. elegans
NIC626 C. elegans
NIC628 C. elegans
NIC630 C. elegans
NIC631 C. elegans
NIC633 C. elegans
NIC636 C. elegans
NIC640 C. elegans
NIC643 C. elegans
NIC646 C. elegans
NIC647 C. elegans
NIC649 C. elegans
NIC650 C. elegans
NIC653 C. elegans
NIC654 C. elegans
NIC655, C. elegans
NIC660 C. elegans
NIC661 C. elegans
NIC662 C. elegans
NIC664 C. elegans
NIC665 C. elegans

NIC666 C. elegans
NIC667 C. elegans
NIC668 C. elegans
NIC671 C. elegans
NIC675 C. elegans
NIC678 C. elegans
NIC680 C. elegans
NIC681 C. elegans
NIC685 C. elegans
NIC686 C. elegans
NIC687 C. elegans
NIC691 C. elegans
NIC692 C. elegans
NIC694 C. elegans
NIC696, C. elegans
NIC697 C. elegans
NIC698 C. elegans
NIC699 C. elegans
NIC700 C. elegans
NIC702 C. elegans
NIC703 C. elegans
NIC704 C. elegans
NIC705 C. elegans
NIC707 C. elegans
NIC709 C. elegans
NIC710 C. elegans
NIC711 C. elegans
NIC713 C. elegans
NIC716 C. elegans
NIC717 C. elegans
NIC721 C. elegans
NIC727 C. elegans
NIC728 C. elegans
NIC729 C. elegans
NIC730 C. elegans
NIC731 C. elegans
NIC734 C. elegans
NIC735 C. elegans
NIC736 C. elegans
NIC739 C. elegans
NIC740 C. elegans
NIC741 C. elegans
NIC742 C. elegans
NIC1671 C. elegans
NIC1672 C. elegans
NIC1673 C. elegans
NIC1675 C. elegans
NIC1676 C. elegans
NIC1697 C. elegans
NIC1701 C. elegans
NIC1702 C. elegans
NIC1713 C. elegans
NIC1714 C. elegans
NIC1715 C. elegans
NIC1716 C. elegans
NIC1717 C. elegans
NIC1719 C. elegans
NIC1720 C. elegans
NIC1723 C. elegans
NIC1724 C. elegans
NIC1725 C. elegans
NIC2091 C. elegans
MY16 C. elegans

Wild animals: N/A

Reporting on sex: Findings apply to hermaphrodites.

Field-collected samples: N/A

Ethics oversight: No ethical approval or guidelines were required because only nematodes were used in this study.

Note that full information on the approval of the study protocol must also be provided in the manuscript.
